# MiR-466 Inhibits the Progression of Severe Hepatocellular Carcinoma via Regulating FMNL2-Mediated Activation of NF-*κ*B and Wnt/*β*-Catenin Pathways

**DOI:** 10.1155/2021/3554219

**Published:** 2021-06-23

**Authors:** Jianwei Li, Su Yan

**Affiliations:** ^1^Department of Hepatology, Traditional Chinese Medicine Hospital of Rizhao City, Rizhao 276800, Shandong, China; ^2^Department of Critical Care Medicine, Traditional Chinese Medicine Hospital of Rizhao City, Rizhao 276800, Shandong, China

## Abstract

Hepatocellular carcinoma (HCC) has threatened the health of humans, and some evidence has indicated that miR-466 involves the progressions of some cancers. This study focused on the role of miR-466 in the formation and development of HCC. The expression levels of miR-466 in the tissues of patients and HCC cell lines were measured by qRT-PCR, and CCK-8, transwell assay, and flow cytometry assay were used to observe the functions of miR-466 on the HCC cells. Moreover, the miRNA databases, dual-luciferase reporter assay, and Western blot were used for the investigation of the regulation mechanism of miR-466 on HCC cells. The results showed that miR-466 was significantly downregulated in HCC tissues and cell lines, and inhibited proliferation, invasion, and high apoptosis were found in HCC cells when miR-466 was overexpressed. The results confirmed that FMNL2 was a target of miR-466, and increased FMNL2 could reverse the effects of miR-466 on the phenotype of HCC cells. Besides, it was also found that miR-466 was involved in the regulation of NF-*κ*B and Wnt/*β*-catenin pathways in HCC cells via targeting FMNL2. In conclusion, the results of this study suggest that miR-466 regulates the activities of NF-*κ*B and Wnt/*β*-catenin pathways to inhibit the progression of HCC cells via targeting FMNL2.

## 1. Introduction

Hepatocellular carcinoma (HCC) is a malignant tumor with high morbidity and mortality, and up to 600000 people die from this disease worldwide every year [1, 2]. Recently, some significant improvements have been achieved in clinical diagnosis and treatment. However, the situations of more than half of patients have been developed to medium or late stage of HCC when they feel obvious discomfort, and the drugs-resistance and tumor invasion can also impede the healing of the patients [3, 4]. Thus, even with current therapeutic strategies, the prognosis and overall survival rates of HCC patients remain unsatisfactory [5, 6]. Although great achievements in the pathogenesis of HCC have been obtained in the last decade, the molecular mechanism on the progression of HCC is not completely illustrated [7, 8]. Therefore, there is an urgent demand to search for novel molecules and provide much more strategies for clinical diagnosis and treatment.

MicroRNAs (miRNAs), the single chain of noncoding RNA with 18–25 nucleotides, are characterized by regulating progressions of gene translations via interacting with the 3′-untranslated regions (3′-UTRs) of the related messenger RNAs (mRNAs) [9]. MiRNAs take part in the signal transmissions and regulate the life activities of cells such as proliferation, differentiation, and apoptosis [10]. Multiple studies have indicated that miRNAs dysfunction plays an important role in the formations and developments of many diseases ranging from cardiovascular deterioration to tumors [11, 12]. Therefore, regulating the expression of some special miRNAs has been proved as a promising diagnostic and therapeutic system for cancer in clinical treatment. The studies have shown that the downregulation of miR-466 is related to the developments of epithelial ovarian cancer and colorectal cancer, while its role in HCC remains unclear [13, 14].

In this study, the exploration attempted to investigate the connection of miR-466 and HCC and reveal the effects of miR-466 on the phenotype of HCC cells and the regulation mechanism of miR-466 on the progression of HCC.

## 2. Materials and Methods

### 2.1. Clinical Tissues

This study was approved by the Ethics Committee of the Traditional Chinese Medicine Hospital of Rizhao, Rizhao, Shandong, China, and the prior consent of the patients and authorizations from hospitals were obtained. The tumor tissues and matched adjacent health tissues donated by the patients were used in this study. All tissues were stored at −80°C.

### 2.2. Cell Culture and Transfection

The normal human liver cell lines HL-7702 and the human hepatic carcinoma cell lines including Hep3B, Huh7, and HepG2 were used in this study. All cells were cultured with Dulbecco's modified eagle medium (DMEM, Procell Life Science and Technology Co., Ltd., China) containing 10% fetal bovine serum (FBS, ThermoFisher, USA) in an incubator with 37°C and 5% CO_2_. The subculture of the cells was performed when the cellular confluence was at 90%.

The cells were seed into the 6-well plates, and cell transfection was performed when the confluences of the cells were at 70%. MiR-466 mimics, miRNA negative control (miR-NC), pcDNA-FMNL2, and pcDNA-NC were synthesized by Generay Biotech Co., Ltd. (Shanghai, China). In short, 4 g of DNA, 100 pmol RNA, or 10 *μ*l lipofectamine 2000 (Beijing Noble Ryder Technology Co. Ltd., Beijing, China) were, respectively, diluted and incubated with 250 *μ*L serum-free medium for 5 min. The diluted DNA or RNA was, respectively, mixed with isometric diluted lipofectamine 2000 and then was incubated at 25°C for 20 min. After that, 500 *μ*L of the mixtures was added into each well, and then, the cells were cultured for 24 hours.

### 2.3. Real-Time Quantitative Reverse Transcription PCR (qRT-PCR)

The miR-466 levels in the tissues and cell lines were measured by qRT-PCR. The TRIzol reagent was used to perform the extractions of the total RNAs in the tissues or HCC cell lines. The concentrations of the total RNAs were measured by spectrophotometry. After that, the total RNAs were transcribed as cDNA via PrimeScript® RT Reagent Kit (Thermo Fisher, Massachusetts, USA). The primers were synthesized and purified by Synbio Technology (Suzhou, China). The reaction systems (10 *μ*L) of qRT-PCR were prepared according to the operational instruction of a KAPA qRT-PCR kit (Sigma-Aldrich, Missouri, USA). U6 was used as the endogenous control. The following conditions were used: denaturation at 95°C for 3 min, followed by amplification for 40 cycles at 95°C for 12 s, at 53°C for 40 s, and 70°C for 30 s. The relative levels of miRNAs were calculated with the 2^−(ΔΔCt)^ method. The primers of miR-466 and U6 were synthesized and purified by RiboBio Co., Ltd. (Guangzhou, China). The primer sequences of miR-466 and U6 are given in Table 1.

### 2.4. Western Blot

The total proteins in the tissues and cell lines were extracted with RIPA buffer on the ice box, and the concentrations of the extractions were measured by a BCA protein assay kit (ThermoFisher, Massachusetts, USA). The extracts were mixed with quadruple of SDS-PAGE sample loading buffer; then, the extracts were boiled at 100°C for 5 min. The proteins were separated by SDS-polyacrylamide gel electrophoresis (SDS-PAGE) and then transferred on the polyvinylidene fluoride (PVDF) membranes by the wet transfer method. After that, the membranes were blocked with 5% fat-free milk at 4°C for 1 hour, and then, membranes were incubated added with the related primary antibodies at 4°C overnight. The membranes were washed with Tris buffered saline-Tween (TBST) for three times (15 mins per time) and then were incubated with second antibodies at 25°C for 1 hour. Finally, the membranes were washed with TBST and added with ECL reagent (ThermoFisher, USA) for three times (10 mins per time). After that, the expressions of the proteins were observed under a chemiluminescence detection system. The antibodies were used under following conditions: anti-FMNL2 (1 : 1000, ab2540720, ThermoFisher, Massachusetts, USA); anti-*β*-catenin (1 : 1000, ab2533039, ThermoFisher, Massachusetts, USA); anti-Wnt (1 : 1000, ab11154198, ThermoFisher, Massachusetts, USA); anti-*P*65 (1 : 1000, ab2533893, ThermoFisher, Massachusetts, USA); anti-*p*-*P*65 (1 : 1000, ab10982265, ThermoFisher, Massachusetts, USA); and anti-*β*-actin (1 : 1000, ab2223496, ThermoFisher, Massachusetts, USA).

### 2.5. Transwell Assay

For the invasion assay, Matrigel was diluted with eight-time DMEM, and diluted Matrigel was added into the upper chambers of transwells. After drying, 5 × 10^4^ cells and 200 *μ*L of serum-free DMEM were added into the upper chambers, and 600 *μ*L of DMEM containing 10% FBS was added into the cells in the lower chambers. The cells were cultured for 24 hours. After that, the cells on the upper surfaces of the chambers were removed by cotton buds, and the migrated cells on the lower surface of the upper chamber were fixed by methanol for 10 min; the cells were then dried at 25°C. The cells were stained with 0.1% w/v crystal violet (Solarbio, Beijing, China) for 30 mins and then washed with tap water. The invaded cells were calculated and photographed under a Leica DMi8 microscope.

### 2.6. CCK-8 Assay

The cells (3 × 10^3^) were seeded into 96-well plates and incubated for 24 hours. After transfection, the cells were further incubated for 24 hours. Subsequently, the viability of the cells at 0, 24, 48, and 72 hours were measured by CCK-8 kit (Amyjet, Wuhan, China). In short, 10 *μ*L of CCK-8 solution was added into each well, and then, the cells were incubated at 25°C in the dark for 4 hours. Finally, the absorbance value of the cells was measured by a microplate reader (Molecular Devices, Shanghai, China).

### 2.7. Dual-Luciferase Reporter Gene Assay

The mutant or wild 3′-UTR sequences of FMNL2 were inserted into the pmirGLO luciferase reporter vectors (Yanjiang Bio Co., Ltd., China) to establish the FMNL2-mutant type (FMNL2-mut) and FMNL2-wild type (FMNL2-wt), respectively. FMNL2-mut or FMNL2-wt was, respectively, cotransfected with miR-466 mimics or miR-NC into HEK-293T cells. After that, the cells were incubated for 48 hours. Finally, the binding effect of miR-466 and FMNL2 was observed by a dual-luciferase reporter assay system.

### 2.8. Flow Cytometry Assay

Huh7 cells were harvested by trypsinase (0.25%, EDTA-free). The harvested cells were washed by 3 mL of ice phosphate-buffered saline (PBS) for once and then were fixed by alcohol. After that, 1 × 10^6^ of the cells were suspended with 100 *μ*l of incubation buffer. 5 *μ*L of ice Annexin V-FITC and 5 *μ*L of propidium iodide (PI 20 *μ*g/ml) were added into the tubes, and then, the cells were incubated in dark for 15 min. Finally, the apoptosis levels of the cells were instantly observed by flow cytometry equipment (BD Biosciences, State of New Jersey, USA).

### 2.9. Statistical Analysis

All experiments were performed at least 3 times, independently. The data were analyzed by SPSS 20.0, and the figures were charted by GraphPad Prism 8.0. The difference of the data was tested with the chi-squared test or ANOVA with Tukey's posthoc test. *P* < 0.05 means that the difference between the two groups was significant.

## 3. Results

### 3.1. MiR-466 was Significantly Downregulated in Tumor Tissues and Cell Lines

**Figure 1:**
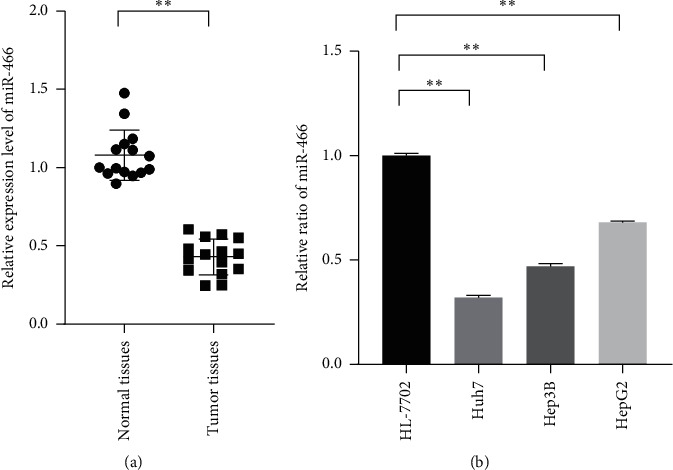
MiR-466 significantly downregulated in HCC tissues and cell lines. (a) The relative expression levels of miR-466 in the tumor and paracancerous tissues measured by qRT-PCR. (b) The relative expression levels of miR-466 normal human liver and HCC cell lines measured by qRT-PCR. ^*∗∗*^, *P* < 0.05.

To observe the connection between miR-466 and hepatocellular carcinoma, the tissues of the patients and normal and tumor cell lines were used to measure the expression level of miR-466. The qRT-PCR showed that the miR-466 was significantly downregulated in the tumor tissues compared with the paracancerous tissues (Figure 1(a), *P* < 0.01). Besides, decreased miR-466 levels were also observed in the tumor cell lines including Hep3B, Huh7, and HepG2 compared with the normal cell line (Figure 1(b), *P* < 0.01).

### 3.2. MiR-466 Inhibited the Proliferation and Invasion of Hepatocellular Carcinoma Cells

**Figure 2:**
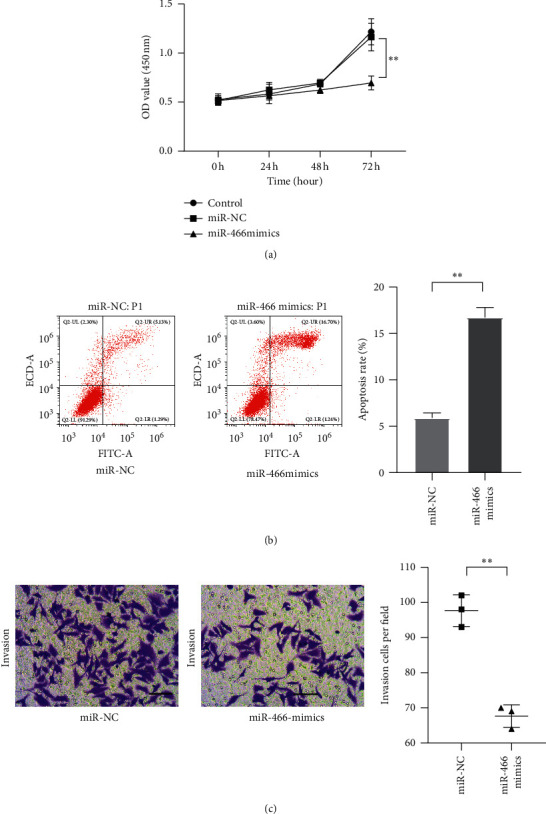
MiR-466 inhibited the proliferation, invasion, and apoptosis of Huh7. (a) The proliferation of Huh7 cells measured by CCK-8. (b) The apoptosis level of Huh7 cells observed by flow cytometry assay. (c) The invasion of Huh7 cells observed by transwell assay (scale bar = 50 *μ*m). ^*∗∗*^, *P* < 0.05.

To explore the functions of miR-466 in hepatocellular carcinoma cells, the miR-466 mimics were transfected into Huh7, and CCK-8 assay, transwell assay, and flow cytometry assay were used to reflect the changes in proliferation, invasion, and apoptosis levels of the cells. The CCK-8 assay showed that the proliferation of Huh7 transfected with miR-466 mimics was visibly inhibited compared with the cells transfected with miR-NC (Figure 2(a), *P* < 0.01). The transwell assay reflected that Huh7 cells transfected with miR-466 mimics expressed low invasive abilities compared with the cells transfected with miR-NC (Figure 2(c), *P* < 0.01). Moreover, the increased apoptosis levels were observed in the cells transfected with miR-466 mimics (Figure 2(b), *P* < 0.01).

### 3.3. MiR-466 Directly Targeted the 3′-UTR of FMNL2

**Figure 3:**
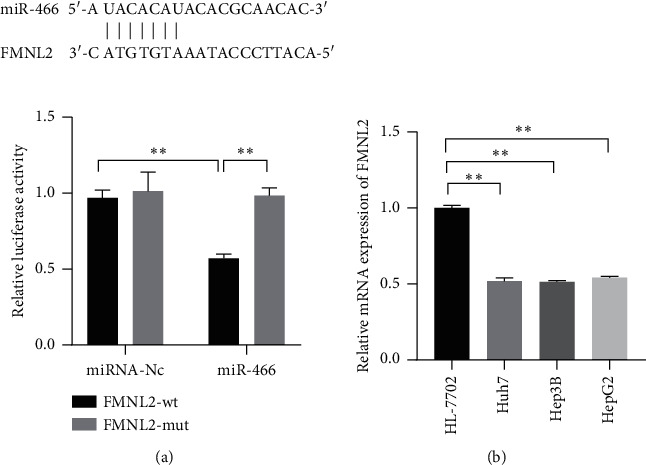
FMNL2 was a downstream target of miR-466 and upregulated in HCC tissues and cell lines. (a) The binding effect of miR-466 and FMNL2 measured by dual-luciferase reporter assay. (b) The expression levels of FMNL2 in HCC cell lines observed by Western blot. ^*∗∗*^, *P* < 0.05.

To reveal the regulation mechanism of miR-466 on HCC, TargetScan, an online database, was used to search the downstream target of miR-466, and a dual-luciferase reporter assay was used to observe the binding effect of miR-466 and its target. The results showed that FMNL2 was one of the potential targets of miR-466. The dual-luciferase reporter assay showed that miR-466 significantly reduced the luciferase activity of the HEK-293T cells transfected with FMNL2 expressed vectors (Figure 3(a), *P* < 0.01). Besides, it was also observed that FMNL2 was extremely upregulated in Hep3B, Huh7, and HepG2 cells compared with normal cell lines (Figure 3(b), *P* < 0.01).

### 3.4. FMNL2 Reversed the Effects of miR-466 on HCC Cells

**Figure 4:**
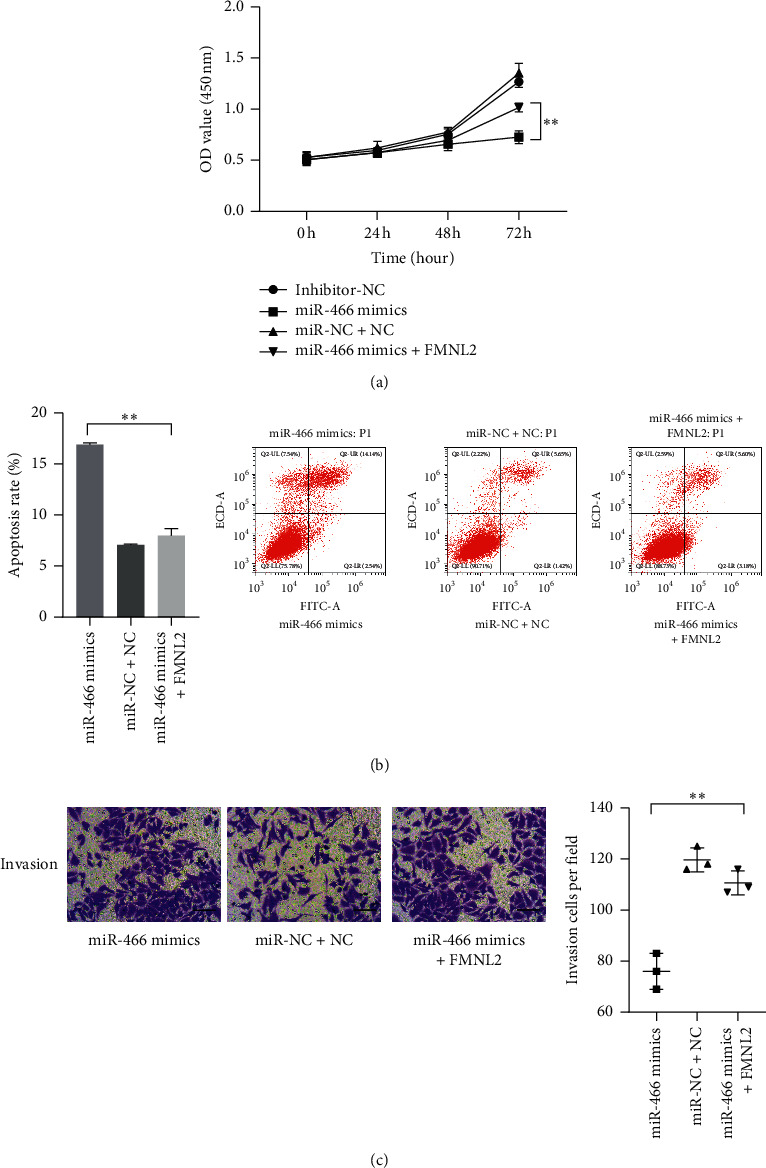
FMNL2 could reverse the effects of miR-466 on the proliferation, invasion, and apoptosis of Huh7. (a) The proliferation of Huh7 cells measured by CCK-8. (b) The apoptosis level of Huh7 cell observed by flow cytometry assay. (c) The invasion of Huh7 cells observed by transwell assay (scale bar = 50 *μ*m). ^*∗∗*^, *P* < 0.05.

Although the connection of miR-466 and FMNL2 was proved above, whether FMNL2 was involved in the regulation of miR-466 on HCC cells remained unclear. The miR-466 mimics and FMNL2 expressed vectors were cotransfected into the HCC cells, and the changes in the proliferation, invasion, and apoptosis of the cells were observed. The CCK-8 assay showed that the weakened proliferation of the cells induced by miR-466 was reversed by FMNL2 upregulation (Figure 4(a), *P* < 0.01). The transwell assay showed that the inhibitory effect of miR-466 on the invasion of the cells was returned by FMNL2 (Figure 4(c), *P* < 0.01). Moreover, it was observed that the increased apoptosis levels of HCC cells including Hep3B, Huh7, and HepG2 induced by miR-466 were rescued by FMNL2 (Figure 4(b), *P* < 0.01). Those observations suggested that miR-466 could regulate the phenotype of the cells via targeting FMNL2.

### 3.5. MiR-466 Inactivated the Wnt/*β*-Catenin and NF-*κ*B via Targeting FMNL2

**Figure 5:**
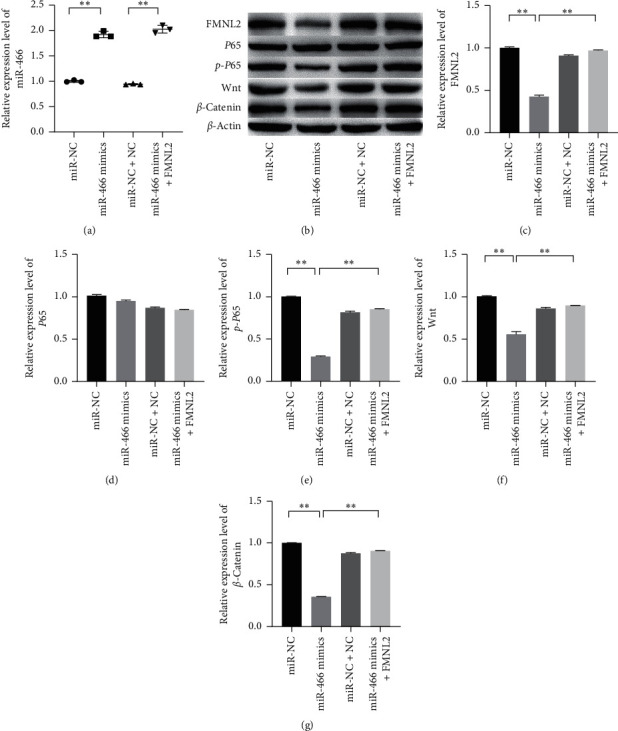
FMNL2 could reverse the effects of miR-466 on NF-*κ*B and Wnt/*β*-catenin pathways in Huh7 cells. (a) The relative expression level of miR-466 tested by qRT-PCR. (b–g) The relative expression levels of FMNL2, *P*65, *p*-*P*65, Wnt, and *β*-catenin measured by Western blot. ^*∗∗*^, *P* < 0.05.

FMNL2 was proved as a key factor in the regulation of miR-466 on HCC. To further analyze the regulation mechanism of miR-466 on HCC, the miR-466 mimics and FMNL2 were cotransfected into the cells, and the proteins in Wnt/*β*-catenin and NF-*κ*B pathways were observed by Western blot. The results showed that miR-466 significantly inhibited the expressions of Wnt, *β*-catenin, and *p*-*P*65. However, the upregulations of those proteins induced by miR-466 could be reversed by FMNL2 (Figure 5, *P* < 0.01).

## 4. Discussion

HCC is one of the malignant diseases which has threatened the health of human, and miRNA dysfunction has been found as a key cause of cancer formation [15]. MiR-466 locates on chromosome 3, and several studies have indicated that miR-466 is significantly downregulated in some tumor cells and involves the regulations of the proliferation and invasion of the tumors [16]. In this study, the expression levels of miR-466 in HCC tissues and cell lines were investigated, and the effects of miR-466 on the phenotype of HCC cells such as proliferation, invasion, and apoptosis were also explored. Subsequently, this study also researched the downstream target of miR-466 and further revealed the role of FMNL2 in HCC progression. Moreover, the effects of miR-466 on the activities of NF-*κ*B and Wnt/*β*-catenin pathways were also illustrated in the present study.

MiRNAs dysfunction is a general event in multiple tumors, and some of them have emerged as keys players in cancer progressions, and intervention on the expressions of some special miRNAs has been considered as a promising strategy for cancer treatment [17, 18]. Hence, the regulation of miRNAs in cancer has been increasingly appreciated [19]. This study determined that miR-466 was significantly downregulated in the HCC tissues and cell lines. MiRNAs contribute to affect the metabolism, resistance, and some other behaviors of tumor cells [20]. The downregulated miR-466 has been found in several cancers, and it plays an inhibitor role to suppress the proliferation and invasion of some tumors [21]. In this study, it was also found that miR-466 upregulation was adverse to the proliferation, invasion, and survival of HCC cells.

The consideration has been given in the character of miRNAs on regulating protein expression via interacting with the related mRNAs in this study. It was found that formin-like protein 2 (FMNL2) was a downstream factor of miR-466, and increased FMNL2 was also observed in HCC tissues and cell lines. FMNL2 is closely related to the formation and development of many tumors, and the carcinogenicity of FMNL2 has been corroborated by several studies. The study has pointed out that FMNL2 is significantly upregulated in colorectal cancer, and FMNL2 silence induced by miR-206 upregulation can visibly inhibit the proliferation and invasion of the tumor cells [22]. In this study, it was testified that increased FMNL2 could also reverse the inhibitory effects of miR-466 on the proliferation, invasion, and survival of the HCC cells.

Molecular mechanisms underlying the growth, invasion, and chemotherapy resistance of HCC have been intensively studied. The aberrant activation of cellular signaling pathways plays a vital role in the deterioration of many types of cancer [23]. One study has indicated that miR-466 is sponged by LINC01152, and its downregulation is related to the dysfunction of the Notch pathway, and miR-466 downregulation indicates the poor prognosis and survival of the patients with glioblastoma multiform [24]. In this study, it was demonstrated that miR-466 upregulation could effectively inactivate the NF-*κ*B and Wnt/*β*-catenin pathways. Aberrant NF-*κ*B and Wnt/*β*-catenin and their downstream factors are widely implicated in numerous malignancies including HCC. A previous study has confirmed that the activated NF-*κ*B induced by casein kinase II subunit beta (CSNK2B) upregulation can promote the unlimited growth and invasion of HCC [25]. This study discovered that miR-466 upregulation is an independent factor to mediate the inactivation of NF-*κ*B and Wnt/*β*-catenin pathways. Moreover, the results also supported that the effects of miR-466 on NF-*κ*B and Wnt/*β*-catenin pathways could be reversed by FMNL2. Yang et al. observed that FMNL2 was significantly upregulated in colorectal cancer cells, and it could further regulate the activation of NF-*κ*B and mediate the invasion of the cancer cells via decreasing the stability of COMMD10 [26]. Moreover, one study has also indicated that FMNL2 could activate the Wnt/*β*-catenin to drive the progression of colorectal cancer [27]. Thus, those observations suggest that increased miR-466 can effectively inactivate the NF-*κ*B and Wnt/*β*-catenin pathways of HCC cells via targeting FMNL2.

This study has revealed the role of miR-466 in the progression of HCC and confirmed the regulation effects of miR-466/FMNL2 on NF-*κ*B and Wnt/*β*-catenin pathways. In summary, it suggests that miR-466 inactivates NF-*κ*B and Wnt/*β*-catenin pathways to inhibit the progression of HCC via targeting FMNL2. However, whether the change of the FMNL2 level is an independent cause of miR-466 on regulating the phenotype and the signal pathways of HCC cells remains unknown. Therefore, more evidence is necessary from the experiments in vivo to corroborate the presumption in this study.

## Figures and Tables

**Table 1:** Primer sequence of miR-466 and U6.

Name of primer	Sequences
miR-466-F	5′-CACTAGTGGTTCCGTTTAGTAG-3′
miR-466-R	5′-TTGTAGTCA CTAGGGCACC-3′
FMNL2-F	5′-GCTATGAACCTACCTCCTGACA-3′
FMNL2-R	5′-AACACGCCGTCTGAATTTCTT-3′
U6–F	5′-CTCGCTTCGGCAGCACA-3′
U6-R	5′-AACGCTTCACGAATTTGCGT-3′

## Data Availability

The data used to support the findings of this study are available from the corresponding author upon request.
